# The Role of NO/sGC/cGMP/PKG Signaling Pathway in Regulation of Platelet Function

**DOI:** 10.3390/cells11223704

**Published:** 2022-11-21

**Authors:** Stepan Gambaryan

**Affiliations:** Sechenov Institute of Evolutionary Physiology and Biochemistry of the Russian Academy of Sciences, 194223 St. Petersburg, Russia; gambaryan@klin-biochem.uni-wuerzburg.de; Tel.: +7-812-552-79-01; Fax: +7-812-552-30-12

**Keywords:** cGMP, nitric oxide, platelet, NOS, PKG

## Abstract

Circulating blood platelets are controlled by stimulatory and inhibitory factors, and a tightly regulated equilibrium between these two opposing processes is essential for normal platelet and vascular function. NO/cGMP/ Protein Kinase G (PKG) pathways play a highly significant role in platelet inhibition, which is supported by a large body of studies and data. This review focused on inconsistent and controversial data of NO/sGC/cGMP/PKG signaling in platelets including sources of NO that activate sGC in platelets, the role of sGC/PKG in platelet inhibition/activation, and the complexity of the regulation of platelet inhibitory mechanisms by cGMP/PKG pathways. In conclusion, we suggest that the recently developed quantitative phosphoproteomic method will be a powerful tool for the analysis of PKG-mediated effects. Analysis of phosphoproteins in PKG-activated platelets will reveal many new PKG substrates. A future detailed analysis of these substrates and their involvement in different platelet inhibitory pathways could be a basis for the development of new antiplatelet drugs that may target only specific aspects of platelet functions.

## 1. Introduction

Platelets have a fundamental role in normal and pathological hemostasis. The fine-tuning between activating and inhibitory factors prevents spontaneous platelet activation/aggregation and occlusion of blood vessels. On the other hand, in the case of vascular injury, platelets adhere to injured endothelium and subendothelial matrix to form localized thrombi and prevent blood loss. Platelets are now considered not only as the main players in hemostasis, but they also play significant roles in inflammation, atherosclerosis, and cancer development. Platelets contain many components that contribute to innate and adaptive immunity. Fc and complement receptors are expressed on the platelet surface, and platelets can migrate to bacterial chemoattractants and generate free radicals and antimicrobial peptides (rev. in refs. [1,2]). The recently discovered platelet C-type lectin-like receptor (CLEC-2) bridges tissue inflammation and vessel thrombosis [3]. Chronic inflammation is one of the primary factors leading to atherosclerosis. Activated platelets and platelet-derived microparticles are important triggers even in the early phase of atherosclerosis [4,5,6].

By activation of corresponding receptors and utilizing multiple intracellular transduction mechanisms, a variety of factors, including collagen, thrombin, ADP, von Willebrand factor (vWF), thromboxane, podoplanin, and others, promote platelet activation. On the other hand, endothelial cell-derived prostacyclin and nitric oxide (NO), by increasing the level of platelet cyclic nucleotides cAMP and cGMP, respectively, represent the major platelet inhibitory machinery. Elevated cAMP and cGMP activate corresponding protein kinases, protein kinase A (PKA), and protein kinase G (PKG), which phosphorylate multiple substrates responsible for platelet inhibition. The mechanisms of PKA/PKG-mediated platelet inhibition have been described in several reviews [7,8,9,10] and the number of identified substrates in platelets phosphorylated by these kinases gradually increased. More than 300 phosphoproteins phosphorylated in response to the cAMP-elevating prostacyclin analogue iloprost were identified by a quantitative phosphoproteomic approach [11]. Among them, 137 contained PKA consensus phosphorylation sites. The same method was applied to platelets in response to cGMP elevation which revealed an increase of phosphorylation of more than 150 proteins and decrease of phosphorylation of more than 60 proteins [10]. In our recent review published in 2018 [10], we presented known mechanisms of cGMP signaling in platelets including regulation of platelet cGMP levels, G-protein coupled receptor (GPCR), calcium signaling, and regulation of platelet small GTPases. The present review focused on some controversial questions concerning sources of NO and activation of soluble guanylate cyclase (sGC) in platelets, role of the NO/sGC/cGMP/PKG pathway in platelets, including new data that were not covered and appeared after publication of our last review concerning PKG substrates.

## 2. Historical Aspects of cGMP Functions in Platelets

Following the discovery and identification of cAMP as the first hormone second messenger by Sutherland and colleagues in 1957/1958 [12], cGMP was first chemically synthesized in 1960 [13] and then identified in rabbit urine as a biologically produced compound [14]. While it was soon established that cAMP mediates the platelet inhibitory effects of certain prostaglandins including prostacyclin [15], the precise role of cGMP remained elusive for some time [16]. Initially, cGMP and cAMP were thought to have opposing roles in biology (the Yin Yang hypothesis), suggesting that platelet activation is inhibited by cAMP and stimulated by cGMP [16].

It took a few years to untangle the contributions of the cGMP in platelets. Some early studies showed that different compounds including collagen [17,18,19], ADP, epinephrine, and arachidonic acid [19,20] that initiate platelet secretion and aggregation elevated cGMP levels. Accordingly, based on these early studies it was hypothesized that cGMP plays a stimulatory role in platelets [17,21,22].

A major and highly influential discovery published in 1977 showed that various smooth muscle relaxants such as nitroprusside, nitric oxide and other nitrovasodilators activate sGC and raise the cellular cGMP level in various tissues [23]. Similar results were subsequently reported also for platelets which indicated that activators of the sGC, including sodium nitroprusside (SNP), sodium azide, nitrosoguanidines, and ascorbic acid markedly increased platelet cGMP levels. In 1980, Haslam [24] proposed that cGMP inhibits platelet activation induced by different stimuli and then proved this hypothesis experimentally [25]. At the same time, expression of cGMP-dependent protein kinase (PKG) and its substrate in platelets was identified and cGMP-dependent platelet inhibition mediated by activation of PKGwas proposed [26]. In numerous papers, the mechanisms of cGMP/PKG-mediated platelet inhibition have been studied and established as summarized in some recent reviews [8,9,27,28]. Thus, the historical view and hypothesis from the 1970ies that cGMP stimulates platelet activation was subsequently corrected by studies from multiple groups and therefore abandoned.

## 3. Current State

### 3.1. NOS/NO in Lymphocytes and Platelets

**Figure 1:**
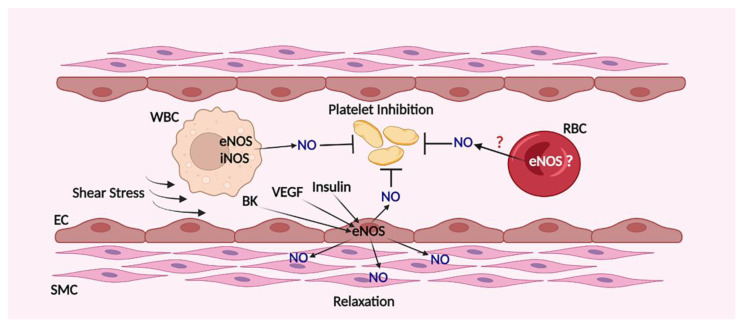
Sources of NO in blood vessels. The main source of NO in blood vessels is endothelial cells that express eNOS, which is activated by different stimuli including shear stress, bradykinin (BK), VEGF, insulin and other stimuli that could increase intracellular calcium concentration. White blood cells (WBC) produced NO by the activity of eNOS and iNOS also involved in platelet inhibition. The questions whether erythrocytes (RBC) also express functionally active eNOS and whether they could produce NO which reaches platelets are still not clear.

Circulating blood cells and endothelial cells are the two main sources of NO that activate platelet sGC. Endothelial cells express eNOS, which is activated by different factors including shear stress, bradykinin, VEGF, insulin, and some others (Figure 1). Different types of lymphocytes express endothelial (eNOS) and/or inducible (iNOS) NO synthase (NOS) and lymphocytes and macrophages start to synthetize NO upon activation. Incubation of lymphocytes with platelets lead to the inhibition of agonist-induced platelet activation mediated by released NO from lymphocytes and the effect was enhanced by activation of iNOS [29,30]. However, the impact of lymphocytes derived NO in circulated blood and its role in activation of platelet sGC in normal and inflammatory conditions remain to be elucidated in more detail.

In the literature there is still no consensus whether eNOS and/or iNOS are expressed in platelets. In other words, whether platelets themselves can produce NO that can act as a feedback inhibitory mechanism to prevent platelet activation is still unknown. NOS/NO-independent activation and increase of cGMP and PKG activity was described in vWF-stimulated platelets [31] and cAMP-independent PKA activation was shown in thrombin- and collagen-stimulated platelets [32]. Both these reactions were proposed as feedback inhibitory signaling mechanisms for preventing undesired platelet activation. In numerous papers, starting from the first indication that platelets themselves produced NO by activation of NOS [33,34], inhibition of platelet activation by NO derived from platelets was shown (rev. in refs. [35,36,37]). In an eNOS knock out (KO) mouse model, platelet-derived NO increased the bleeding time and altered in vivo hemostatic response by increasing platelet recruitment [38]. Several mechanisms including elevation of intracellular calcium concentration, phosphorylation by PKA, PKB, AMPK, tyrosine de-phosphorylation, and acetylation of eNOS were shown to be responsible for eNOS activation and NO production in platelets mediated by different compounds (rev in refs. [39]). In 2017, an interesting paper appeared, where the authors divided human platelets into two distinct populations, one of which expresses functionally active eNOS, and the other one is an eNOS negative population [40]. These two populations were identified by NO specific dye DAF-FM and eNOS expression was detected by antibodies. The fact that circulating platelets are heterogeneous in age, size, receptor expression, and response to the same agonist and that this heterogeneity is probably reflecting differences in megakaryocytes is well known. Three major platelet populations with distinct localization in the arterial thrombi were identified. One of them has high level of integrin αIIbβ3 activation, another with strong surface expression of P-selectin, and phosphatidylserine (PS) expressing procoagulant platelets [41,42,43]. Therefore, it could be possible that the platelets are also heterogeneous by eNOS expression. In the whole platelet population, the authors identified more than 80% eNOS positive platelets that may represent functionally distinct platelet subpopulations and claimed that eNOS positive and eNOS negative populations play differential roles in adhesion and aggregation. In another recently published paper [44], sex-dependent differences in platelet NO production and platelet response to NO derived from platelets was described. The authors found that eNOS expression in healthy women platelets was several times greater than in men and came to the conclusion that “the response of elevated platelet NO in men was increased platelet reactivity and the response of markedly elevated platelet NO in women slightly inhibited platelet reactivity”. These interesting and important data definitely merit future validations and confirmations.

On the other hand, there are also doubts concerning NOS expression and NO production by platelets [31,45,46,47,48] and by most sensitive proteomic and transcriptomic methods neither eNOS nor iNOS protein or mRNA were found in human and mouse platelets [49,50,51]. The dissensions of these data might be partly explained by specificity of eNOS and phospho-eNOS antibodies, some problems of measuring cGMP concentration in platelets, and pitfalls with the measurement of NOS activity (rev. in ref. [39]). However, the questions whether NOS expressed in distinct platelet populations and whether platelets themselves can produce biologically active NO remain open and new methods and experimental approaches should be used to solve this problem.

### 3.2. Erythrocytes as Sources of NO in Blood

The question of whether red blood cells (RBC) express functionally active NOS was first raised in 1998 when the expression of functionally active eNOS was identified in human RBCs [52]. Two years later, the expression of eNOS and iNOS in RBCs was shown by immunoblotting; however, the authors especially emphasized that both NOS isoforms are inactive and do not produce NO [52]. In 2012, new evidence was presented that RBCs express functionally active eNOS [53,54,55] and at the same time doubts appeared concerning functionally active NOS expression in RBCs [56]. In mouse models with endothelial cells and RBCs specific eNOS KO the differential contribution of both systems in blood pressure regulation was shown and the authors concluded that RBCs eNOS represent an independent pathway for regulation of NO metabolism, blood pressure, and systemic hemodynamics [55]. Interestingly, the problem of NOS expression in RBCs is similar to platelet NOS, in both cases expression of any NOS isoforms was not detected by the quantitative proteomic method, the most sensitive method for analysis of protein expression [57].

Another important question, whether RBCs can produce functionally active NO and whether this NO can be involved in platelet inhibition, is highly controversial and very important in clinical aspects. From clinical practice and different experimental models the direct correlation between RBCs count in blood and bleeding time has been known for a long time. Low RBCs levels are directly associated with increased bleeding time, irrespective of platelet count, high hematocrit often correlates with thrombotic events [58], and platelet reactivity increases after RBC transfusion [59]. Direct or indirect interactions of RBCs with platelets and their role in hemostasis is another important question. Recently, elevated GPIbα mediated platelet adhesion rates by binding with VWF in a hematocrit-dependent manner were shown [60]. Activated platelets expose FasL which binds to FasL receptor (FasLR) in RBCs and facilitated PS surface exposure in RBCs and platelets promoting thrombin generation and thrombus formation [61]. All these data suggested that RBCs themselves could play a significant role in hemostasis by activation of platelets [62,63].

On the other hand, platelet inhibition by RBCs derived NO was described in several papers [64,65,66,67,68]. Platelet inhibition by NO should include activation of sGC/cGMP/PKG pathway; however, in all these papers, it was not directly shown that RBCs can activate platelet sGC. To clarify these questions, we used our highly sensitive and well-established method to analyze sGC/cGMP/PKG pathway based on VASP phosphorylation in platelets and in vitro activation of recombinant sGC. We performed experiments in whole blood, and on platelets coincubated with RBCs and concluded that RBCs themselves, in all tested conditions, could not activate platelet sGC; instead, they always acted as strong NO scavengers. Similar results were obtained in in vitro experiments with recombinant purified sGC where addition of RBCs only strongly inhibited cGMP formation [69]. Numerous papers (summarized in the recent reviews [70,71]) are dedicated to the regulation of blood flow and hypoxic vasodilation by RBCs-derived NO; however, the mechanisms of NO formation by RBCs are still under debate in the literature. Two main mechanisms are proposed for this reaction: the first one is connected with nitrite reductase activity of deoxyhemoglobin [72,73], and the second one is mediated by nitrosylation of conserved cysteine on the β-chain of hemoglobin to form SNO-Hb, which can transmit NO out of the RBCs [74,75]. However, others later criticized both these mechanisms [76,77,78] and the question of how RBCs transmit NO to vascular smooth muscle cells and platelets still remains open.

RBCs and platelets represent the highest population in the circulated blood cells and are both involved in thrombus formation and cardiovascular complications in pathological conditions. Therefore, the question of their interaction and especially concerning NO pathways in regulation of platelet function is highly clinically important and merits future investigations.

## 4. Nitrate/Nitrite/NO Pathway

For a long time, dietary nitrate has been recognized as an important precursor of NO independent from NOS. Nitrate is transported by saliva into the salivary glands and secreted into the oral cavity, where it is reduced to the nitrite by specific reductase enzymes of commensal bacteria in the oral microbiota. The one-electron reduction of nitrite to NO occurs by two independent pathways, namely by enzymatic and chemical reactions. Variety of metal containing enzymes and proteins including heme-associated globins, mitochondrial proteins, molybdenum metalloenzymes, and NOS themselves are able to reduce nitrite to NO. By increasing bioavailability of NO, the nitrate/nitrite pathway has beneficial effects on many pathological states including cardiovascular complications (rev in [79,80,81]). However, the process of nitrite reduction to NO in the bloodstream is not fully defined. In numerous publications hemoglobin in RBCs is considered as the main protein that acts as nitrite reductase and that NO released from RBCs is involved in reduced platelet reactivity [80]. However, in experiments with washed platelets and recombinant sGC incubated with RBCs and nitrite neither platelet sGC, nor purified recombinant sGC were activated [69]. Incubation of washed platelets with inorganic nitrite did not significantly activate platelet sGC but it potentiated effects of sildenafil [82]. Carbonic anhydrase (CA) in certain conditions can probably act as a nitrite reductase [83]. Both CA1 and CA2 are expressed in platelets in a relatively high level (2500 and 36,100 copies/platelet respectively) [84]; however, it is not clear whether platelet CA can reduce nitrite to NO. sGC is strongly activated in washed human platelets incubated with recombinant bovine CA2 together with nitrite [83], whereas others showed that human, bovine, or mouse CA2 does not regulate nitrite-dependent NO formation in blood [85]. Recently in experiments with washed human platelets, we showed that cysteine is required for nitrite-dependent sGC activation, which was independent from CA inhibitor [86], but this assumption needs future experimental confirmations. Certainly, different experimental conditions including washed platelets, whole blood, or animal/human experiments could lead to different and even controversial results, but this does not call into question that nitrate/nitrite/NO pathway plays a beneficial role in the cardiovascular system and in regulation of platelet reactivity. However, the question concerning the precise mechanisms of NO synthesis from nitrite in the circulating blood under different conditions and precise role of enzymes with nitrite reductase activity in this process needs future experimental conformations.

### 4.1. Analysis of sGC Activation in Platelets

Particulate guanylate cyclases, activated by natriuretic peptides (NP), and sGC represent two main systems of cGMP synthesis in the cells. NPs, atrial natriuretic peptide (ANP), brain natriuretic peptide (BNP), and C-type natriuretic peptide (CNP) bind to the respective receptors GC-A, GC-B, and GC-C [87]. Natriuretic peptide receptors mRNA were not detected in human platelets and they did not respond to NPs by increase of cGMP or VASP phosphorylation [88]. In a transgenic mouse model, expressing cGMP sensor, infusion of NPs did not induce elevation of cGMP in platelets [89]. Thus, sGC is the only enzyme responsible for cGMP synthesis in platelets. In platelets, only α1 and β1 isoforms of sGC are expressed [90] in equimolar (3700 copies for β1 and 3500 copies for α1 per platelet) concentrations [84].

Activation of sGC and subsequent cGMP accrual was demonstrated in the mouse model expressing cGMP sensor which allows monitoring dynamic changes of the cGMP concentration in intact platelets [89]. In this manuscript, some important data concerning regulation of platelet sGC activation are presented. Mechanistically, it was shown that sGC activation is strongly dependent on the shear stress. However, there are differences between experiments in flow chamber and in vivo model of thrombus formation in the arterioles of the cremaster muscle. In the flow chamber with a collagen-coated surface, cGMP elevation without implication of NO donors was not detected on the growing thrombi, whereas in an in vivo model, strong cGMP accrual was observed and this increase of cGMP was absent in sGC KO mouse. The cGMP signal was stronger in the periphery of the growing thrombi, where platelets are exposed to high shear stress, than in the core region. However, the question whether shear stress induced sGC activation in platelets is mediated by NO (derived from platelets, or other blood cells, or endothelial cells) or by NO-independent sGC activation still remains open.

In global and platelet-specific sGC KO mouse models [90,91], it was clearly shown that sGC in platelets plays only an inhibitory role. However, Zhang et al. [92] using the same platelet specific sGC KO mouse model claimed that sGC plays a dual (stimulatory and inhibitory) role in platelets, but their results were not reproducible by others [93], and also were inconsistent with their own previous papers. In previous papers [94,95,96], thrombin and collagen induced increase of cGMP in mouse platelets only from 2 to maximum 3.5-fold compared with the control. Whereas in ref. [92], thrombin increased cGMP in mouse platelets more than 13-fold, and collagen more than 7-fold. In our experiments, thrombin never increased cGMP levels, but slightly decreased basal VASP phosphorylation at Ser239 [93,97]. We [93,98] and others [91] have used the same platelet-specific sGC and global sGC KO mouse and, in accordance to many other publications, detected exclusively inhibitory effects of sGC and the resulting cGMP in platelets.

Genetic alterations of GUCY1A3 gene coding α1 subunit of the sGC in humans also confirm the inhibitory role of sGC in platelets. The loss of sGC activity caused by several mutations of this gene is linked to an increased risk of hypertension, coronary artery disease, atherosclerosis, moyamoya, achalasia, and myocardial infarction [99,100,101]. Platelets play an important role in all these diseases and impaired platelet inhibition could be one of the consequences of these diseases. Platelets from individuals with mutation of GUCY1A3 gene displayed reduced NO-induced platelet inhibition and increased risk of myocardial infarction [99]. From the other side, opposite rare genetic variants of genes encoding sGCα1 and eNOS which enhanced NO signaling are associated with a reduced risk of coronary and peripheral artery disease and stroke [102].

Based on numerous experimental and genetic literature, we can conclude that sGC plays an exclusively inhibitory role in platelets and that the precise mechanisms and functional roles of human platelet sGC and cGMP is of considerable pharmacological and medical importance considering the wide use of drugs which affect platelet cGMP levels.

### 4.2. Regulation of Platelet cGMP Level by Phosphodiesterases

In the human genome, twenty-one genes encode phosphodiesterases (PDEs). By their specific activity, PDEs can be divided into three groups, ones which specifically hydrolyze only cAMP (PDEs 4, 7, and 8), ones which specifically hydrolyze cGMP (PDEs 5, 6, 9), and ones which hydrolyze both cAMP and cGMP (PDEs 1, 2, 3, 10, 11) [103]. From 11 PDE families, in platelets, by proteomic methods [84], Western blots [104], and PDE activity assays (rev. in ref. [27]), only PDE2A, PDE3A, and PDE5A were identified. PDE2A (cGMP-stimulated PDE) and PDE3A (cGMP-inhibited PDE) are mainly responsible for the regulation of cAMP content in platelets. PDE2 is stimulated by binding of cGMP to regulatory site and hydrolyzes both cGMP and cAMP with similar affinities. PDE3 is inhibited by cGMP and preferentially hydrolyzes cAMP. Concentrations of all three PDEs in platelets were measured by two different approaches: proteomic and Western blot methods with recombinant PDEs proteins as a standard [84,104]. Although there are differences in the absolute values, the relative amount of PDE5A is 8 times more than PDE3A and 22 times more than PDE2A. These data are consistent with the previous finding that the total PDE activity for cGMP is significantly higher than for cAMP [27]. Elevated cGMP concentrations in platelets after stimulation with NO donors rapidly (during 40 s) decrease close to the basal level due to strong activation of PDE5, which correlates with PDE5 phosphorylation by PKG [105]. In contrast, cAMP concentration after stimulation with iloprost or forskolin reaches a plateau and does not decrease then even after 20 min. In PDE5 KO mouse platelets aggregation, ATP release, P-selectin expression, and integrin aIIbb3 activation were significantly inhibited [104,106]. Understanding the mechanisms of PDE5 inhibitors on platelet functions became important after the selective PDE5 inhibitor sildenafil was established as a drug for the treatment of pulmonary hypertension and male erectile dysfunction. This is very relevant since there are case reports of bleeding disorders after sildenafil treatment in men [107].

PDE2 and PDE3 seem to play minor, if any, roles in the regulation of platelet cGMP content and preferentially hydrolyze cAMP [108]. Regulation of platelet cAMP by PDE2 and PDE3 activity is still a controversial topic. Dependent on animal models or inhibitors used, elevated cGMP could differently change cAMP concentration (rev in ref. [27]). The maximal increase of cAMP in human platelets incubated with SNP was less than 50% [109], which is clearly close to the limit of cyclic nucleotide detection systems errors. Incubation of platelets with sGC stimulator riociguat significantly increase cGMP production up to 30-fold above control level, but cAMP concentration was not affected [110]. However, very low or no changes in global platelet cAMP level by cGMP elevation do not exclude that in some specific platelet compartments the activity of PDE2 and/or PDE3 could locally influence cAMP concentration and activate PKA. PDE-mediated compartmentation plays a significant role in cAMP/cGMP signaling in different cell types. In smooth muscle cells [111] and cardiac myocytes [112,113] cAMP/cGMP concentration is tightly regulated in different compartments (microdomains), which is important for the precise spatial and temporal control of cyclic nucleotide signaling in these cells. The question of whether similar mechanisms of cyclic nucleotide compartmentalization are also present in platelets remains open, and merits future experimental confirmations.

### 4.3. Activation of PKG Stimulate or Inhibit Platelets

**Figure 2:**
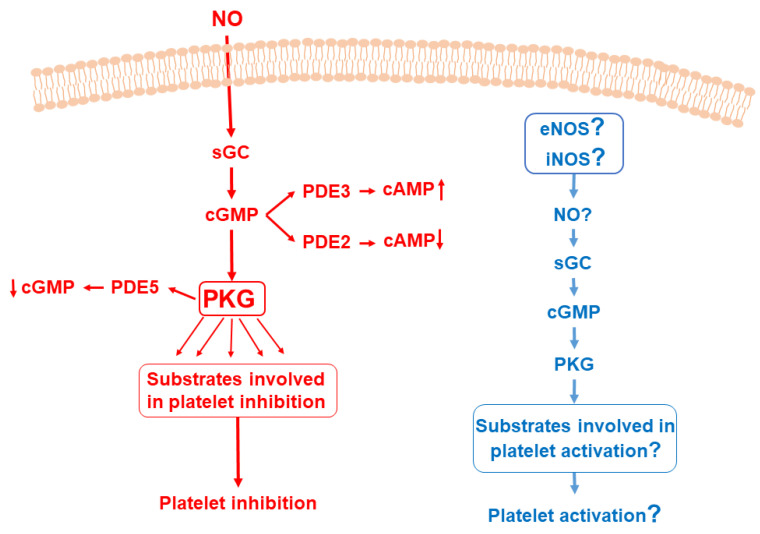
NO/sGC/cGMP/PKG pathways in platelets. (Part in red). Activation of sGC by endogenous NO increases cGMP synthesis. cGMP is involved in regulation of cAMP concentration by PDE2 and PDE3 activation. PDE5 is the main PDE responsible for cGMP degradation. Activated PKG leads to changes in phosphorylation status of numerous proteins and some of them (VASP, IRAG, Rap1B, Rap1Gap2, GRP2, IP3 receptor and others, for details see Section 3) are involved in platelet inhibitory pathways. An alternative hypothesis is presented in blue. However, the expression of any NOS isoform and whether platelets themselves could produce biologically active NO are still under question. In addition, until now, possible involvement of any PKG substrate in platelet stimulatory pathways was not shown (see Section 4.3).

After 1980, for more than 20 years, there was a clear consensus in the platelet literature that cGMP/PKG, in addition to cAMP/PKA pathway, has an exclusively inhibitory role in platelets. However, in 2003, the group of X. Du revisited the historical hypothesis that cGMP/PKG pathway plays a stimulatory role in platelets [114,115] and then this hypothesis was developed in future publications [94,116,117,118,119,120,121] (summarized in Figure 2). The “PKG stimulatory theory” is based on data obtained with the PKG KO mouse model, which directly contradicted the first paper on platelet function in this model [122]. Massberg et al. showed that PKG (referred there as cGKI) only has inhibitory roles in platelets stimulated with collagen or thrombin, or in the model of Ischemia/Reperfusion (I/R).

In 2004, two accompanying papers [123,124] and a letter to the editor [125] published in *Blood* documented that key experiments (including PKG KO mouse model) used as basis for the “PKG stimulatory” concept could not be reproduced and contained methodological errors. However, already in the published response [116] and subsequent publications [116,117,118,119], these arguments were not taken into account and the development of the “PKG stimulatory” concept continued.

Three main important points (in addition to PKG KO mouse model) regarding why the experimental basis for the “PKG stimulatory” concept has important flaws are given as follows. First, the PKG inhibitor (KT5823), which was used to inhibit and therefore prove PKG effects, is not specific [126] and does not inhibit PKG in intact platelets and other cell types [127,128]. Second, cGMP analogs, stimulators (8-Br-cGMP, 8-pCPT-cGMP, 8-Br-PET-cGMP), as well as inhibitors (Rp-8-PCPT-cGMPS, Rp-8-Br-PET-cGMPS) can inhibit or stimulate platelets unrelated to PKG action [123,124,129,130]. Biphasic cGMP effect on platelets (fast activation, then inhibition after 10 min of incubation with cGMP analogs) could be explained by permeability of the cyclic nucleotide analogs. The analogs need at least 10 min to reach the concentration sufficient for platelet PKG activation [123,130,131]. Therefore, the initial stimulatory effect is mediated by unspecific (cGMP/PKG-independent) platelet activation and the subsequent inhibitory action represents the real cGMP/PKG-dependent platelet inhibition. Third, a sequential activation of p38 and ERK/MAP kinases by PKG was described as molecular mechanisms of the “PKG stimulatory” concept. Importantly, from 2003 until now no other laboratory has been able to reproduce these signaling data, and many publications have documented opposite effects, i.e., inhibition of MAP kinases by PKG [123,124,132,133,134]. In addition, the platelet stimulatory effects of sildenafil [115] could not be reproduced by many laboratories and experts in the field, and in many papers sildenafil was shown to have only inhibitory effects on platelets and especially strong potentiation of platelet inhibition induced by NO-elevating compounds [82,135,136,137,138].

The question of whether sGC/cGMP/PKG pathways have an inhibitory (as documented in numerous studies) or stimulatory role in platelets is fundamental for medical practice considering the wide use of drugs which affect platelet cGMP levels. In the literature, there are no indications that nitrovasodilators, NO donors, sGC stimulators, or PDE inhibitors could activate human platelets or induce thrombotic complications. Therefore, the postulation that sGC/cGMP/PKG pathway plays a stimulatory role in platelets, similar to historical view on this problem (see Section 1), could be abandoned.

### 4.4. PKG Activation Inhibited Pro-Coagulant Platelet Formation But Not Platelet Apoptosis

**Figure 3:**
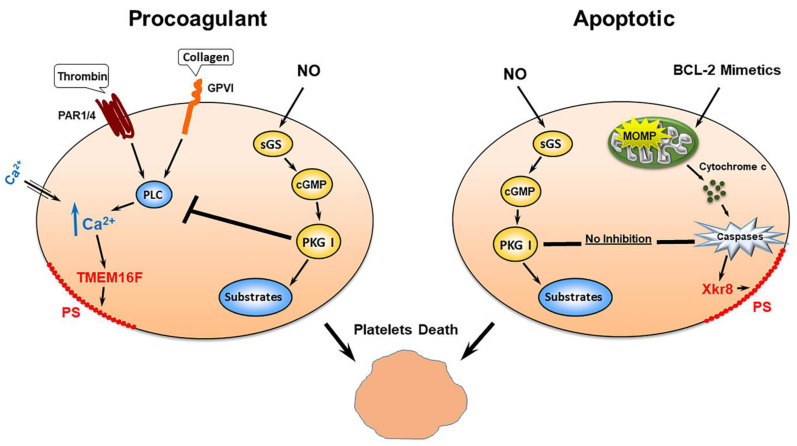
PKG activation inhibits pro-coagulant platelet formation but not platelet apoptosis. Both procoagulant and apoptotic platelets formation at the end lead to platelet death. Procoagulant platelets are formed by strong agonists (thrombin plus collagen) that activate respective receptors which leads to sustain increase of intracellular calcium concentration and activation of TMEM16F and PS surface externalization. Activation of PKG by endogenous NO by different mechanisms inhibited increase of intracellular calcium concentration and procoagulant platelet formation. Bcl-2 mimetics induce platelet apoptosis by activation of caspases pathways; however, in contrast to procoagulant platelets, activation of PKG does not inhibit platelet apoptosis.

Platelet transformation, apoptosis, and formation of procoagulant platelets are mediated by two independent mechanisms. Some common features including strong phosphatidylserine (PS) surface exposure, loss of mitochondrial membrane potential, and microparticle formation characterize both these states. The main differences between mechanisms that trigger platelet apoptosis and procoagulant platelet formation are connected with caspase activation. Platelet apoptosis is mediated by strong caspase activation, whereas procoagulant platelet formation is caspase-independent [139]. In apoptotic and pro-coagulant platelets, surface PS exposure is mediated by different pathways. In pro-coagulant platelets, PS exposure is triggered by activation of calcium dependent scramblase transmembrane protein 16F (TMEM16F), whereas in caspase-dependent apoptotic platelets it is mediated by Xk-related protein family member (Xkr8) [140,141] (summarized in Figure 3). Generally, procoagulant platelets are formed during strong activation (usually a combination of thrombin and collagen induces this stage) and triggered by high sustained intracellular calcium level [139,142,143]. The frmation of pro-coagulant platelets is an irreversible process which ultimately leads to platelet death and this process is independent from apoptosis and caspase activation pathways [139]. Several proteins involved in apoptosis machinery are expressed in platelets and an intrinsic program for apoptosis controls platelet survival and their life span [144]. Only limited information concerning regulation of apoptosis and pro-coagulant platelet formation by cyclic nucleotides is available in the literature. PKA activity protects platelets from apoptosis by different mechanisms including phosphorylation of BAD [145,146]. Importantly, we found that a potent Bcl-2 mimetic, ABT-737 induced caspase-dependent apoptosis which was accompanied with strong cAMP-independent PKA activation and VASP phosphorylation. However, in our experiments, pre-activation of PKA or PKG did not inhibit caspase-dependent platelet apoptosis. In contrast, the activation of both PKA and PKG strongly inhibited procoagulant platelet formation [147].

Using a platelet-specific sGC KO mouse model, we showed that inhibition of procoagulant platelet formation by NO is mediated by cGMP-dependent and -independent mechanisms. Inhibition of PS surface exposure is mediated only by the activation of the sGC/PKG pathway. At the same time, changes of mitochondrial membrane potential were dependent on NO concentration; at low NO concentration it was mediated by the sGC/PKG pathway and at high NO concentration it was cGMP-independent [98]. Similarly, in nucleated cells NO is also involved in the regulation of pro- and anti-apoptotic reactions by cGMP-dependent and-independent mechanisms [148,149,150]. All these data indicate that regulation of apoptosis and procoagulant platelet-formation processes by NO are very complex and merit future investigation including novel proteomic approaches by the analysis of protein S-nitrosylation of cysteine, nitration of tyrosine residues, and phosphorylation of PKG substrates during platelet transformation.

### 4.5. Regulation of Megakaryocyte Function by NO/cGMP/PKG Pathway

Platelet production from megakaryocytes is a complex process that includes megakaryocyte polyploidization, maturation, and platelet production. The question of whether and how the NO/cGMP/PKG pathway is involved in regulation of these processes is not clear yet and strongly dependent on experimental procedure and sources of megakaryocytes. Megakaryocytes are localized in the vascular niche in close proximity to sinusoid endothelial cells [151,152] and endothelial cells derived NO can affect their maturation and platelet production. A significant increase in the platelet number was detected in global PKG1 KO mouse; however, the fact that in platelet specific PKG1 KO mouse platelet count did not differ from wild mouse indicated that thrombocytosis is not mediated by PKG function in megakaryocytes. Furthermore, thrombocytosis in the global PKG1 KO mouse was associated with enhanced interleukin-6 production from nonhematopoietic cells [153]. Platelet count in eNOS KO mouse did not differ from WT mouse and was significantly lower in iNOS KO mouse [154], but there were no differences in platelet aggregation in response to thrombin on these mouse models [31].

Several in vitro models were used for analysis of the NO/cGMP/PKG pathway on megakaryocytes derived from different sources. In Meg-01 megakaryocytic cell line NO induced apoptosis [155] and facilitated platelet-sized particle production [154]; however, it was not analyzed whether the cGMP/PKG pathway is involved in these processes. CD34+ hematopoietic progenitor cells (HPC) isolated from healthy donors, from patients with different tumors, and from chronic myelocytic leukemia (CML) patients were used for the analysis of the cGMP/PKG pathway’s effects on proliferation and differentiation of these cells to megakaryocytes [156]. Stimulation of PKG by cell-permeable cGMP analog (8-pCPT-cGMP) differentially affected HPC proliferation and differentiation into megakaryocytes. Low concentration (25 µM) inhibited proliferation and stimulated differentiation of these cells, whereas high concentration (100 µM) stimulated proliferation and inhibited differentiation to megakaryocytes (CD41+ cells) in control and tumor patients. There were no consistent results of PKG stimulation on cells isolated from four CML patients. In the same experimental setting, HPC from each patient reacted differentially (no effect or stimulation/inhibition of proliferation or differentiation) on PKG activation. Importantly, PKG expression during 10 days of HPC culture was dramatically down-regulated [157], and therefore these results definitely did not reflect in vivo situations. The opposite situation concerning PKG expression was described in the model of megakaryocyte differentiation from mouse fetal liver cells (FLC). Expression of sGC, PKG, and its major substrate VASP was strongly up-regulated at day 4 during differentiation of FLC to mature megakaryocytes. cGMP concentration significantly increased in megakaryocytes at second day of FLC culture and returned to the level FLC at fourth day of culture and it correlated with up-regulation of PDE5 expression. Strong differences were observed between cAMP/PKA and cGMP/PKG signaling; PKA activation promoted megakaryocyte differentiation and suppressed platelet production, whereas cGMP/PKG signaling did not affect megakaryocyte differentiation, but significantly stimulated platelet production [158].

Presented data showed that cell culture models for analysis of cGMP/PKG system in megakaryocyte function have strong limitations and do not reflect real in vivo situations. It seems that, at least in mouse, according to the PKG1 KO model, PKG is not involved in the regulation of megakaryocyte function; however, this does not exclude different situation in humans. The analysis of platelet count and megakaryocytes from some patients taking sGC stimulators/activators and individuals with mutations of the GUCY1A3 gene probably could help to answer these questions.

## 5. PKG Substrates in Platelets

In our recent review [10], we presented an analysis of cGMP/PKG-mediated effects on platelets with a special emphasis on inhibition of calcium signaling, G-protein coupled receptors, and small GTPases. This chapter is focused on the functional significance of some new PKG substrates identified by phosphoproteomic analysis in our ongoing project.

PKG-dependent inhibition of platelets is mediated by the phosphorylation of target proteins (substrates) that are involved in diverse intracellular reactions. Before the introduction of quantitative phosphoproteomic methods only a few (less than 15) substrates were identified in platelets by radioactive methods (32P incorporation into certain proteins) and phospho-specific antibodies. In our recent review [10], we partly presented data of our ongoing project on analysis of PKG specific substrates in platelets. The increase of phosphorylation of more than 150 proteins and decrease of phosphorylation of more than 60 proteins were identified by quantitative phosphoproteomic approaches. In a more detailed analysis of sGC activator (riociguat)-stimulated platelets, 8181 phosphorylation sites from 2249 proteins were quantified. Among them, in 345 proteins phosphorylation levels increased (more than 1.5-fold) and decreased (more than 1.5-fold) in 94 proteins. PKG phosphorylated proteins are involved in almost all platelet functions including membrane proteins and receptors, regulation of cytoskeleton activity, platelet granule proteins and those involved in granule release, and in numerous signaling pathways including several protein kinases and phosphatases [159]. Not all proteins phosphorylated by PKG activation contain canonical PKG phosphorylation sites (R/K|R/K|X|S/T), indicating that the activity of other kinases and phosphatases is regulated by PKG activation. A quantitative phosphoproteomic analysis of PKG-stimulated platelets revealed a complex network of signaling molecules and future validation of phosphorylated proteins by different methods and analysis of their involvement in platelet functions is an important future task. In this respect, some of the newly identified PKG substrates including ENSA/ARPP19 and proteins involved in small GTPase RhoA regulation, RhoA-specific GTPase-activating protein Myo9b, and guanine nucleotide-exchange factor GEF-H1 were analyzed. PKG and PKA have overlapping phosphorylation consensus sequences (R/K|R/K|X|S/T) and these proteins were phosphorylated by both kinases. ENSA/ARPP19 are important regulators of protein phosphatase 2A (PP2A). Microtubule-associated serine/threonine kinase-like (MASTL) phosphorylate ENSA at S67 and ARPP19 at S62 and phosphorylation of these sites converts both proteins into PP2A inhibitors. Inhibition of PP2a strongly prevents thrombin-induced platelet aggregation and increases phosphorylation of several proteins. [160]. Activation of PKA/PKG induces strong phosphorylation of ENSA at S109 and ARPP19 at S104 with concomitant reduction of S67 (ENSA) and S62 (ARPP19) phosphorylation, which reduces PP2A activity [161]. In a MASTL KO mouse model, platelet abnormalities were associated with inhibition of PP2A [162] and our data indicated a strong crosstalk between PKA/PKG and phosphatase activity in platelets.

RhoA in platelets plays an important role in cytoskeletal rearrangements by regulation of actin contractility and contributes to platelet shape change reaction, spreading, thrombus stability and reactive oxygen species (ROS) generation [163,164]. RhoA activation is strongly inhibited by PKA and PKG; however, the detailed mechanisms of this inhibition are still under debate and are controversial. The direct phosphorylation of RhoA at S188 by PKA and PKG was proposed as one of the main mechanisms of RhoA inhibition by these kinases [165,166,167]. On the other hand, it was shown that in platelets spread on fibrinogen-coated surface RhoA were phosphorylated by PKA but not by PKG [168] and the authors concluded that activation of PKA and PKG can synergistically and independently regulate RhoA activity and actin cytoskeleton. Interestingly, direct RhoA phosphorylation at S188 was not detected by phosphoproteomic analysis of PKA/PKG-stimulated platelets [10,11,169] indicating that probably direct RhoA phosphorylation might be connected with specificity of the phospho antibodies. A similar problem connected with antibody specificity was discussed above for eNOS [31]. Several GTPase-activating proteins (GAPs) and guanine nucleotide-exchange factors (GEFs) control the activity of small GTPases and some of them are known substrates for PKA/PKG [170,171,172]. Recently, RhoA-specific GAP (Myo9b) and GEF (GEF-H1) were found to be substrates for PKA/PKG and phosphorylation of Myo9b at S1354 and GEF-H1 at S886, but not phosphorylation of RhoA itself, is involved in the regulation of RhoA activity [173].

These data indicate the complexity of molecular mechanisms involved in regulation of platelet inhibitory pathways by PKA/PKG and future analysis of how the identified substrates of these kinases are involved in regulation of platelet function by different approaches including bioinformatics, modeling, molecular biology, etc. could shed new light on platelet biology and help to solve many contradictory questions concerning the function of the NO/sGC/cGMP/PKG pathway in platelets.

## 6. Conclusions and Future Perspectives

The main goal of this review was to show the fine-tuning and complexity of the regulation of platelet inhibitory mechanisms by the cGMP/PKG pathway with a special emphasis on controversial literature data of NO/sGC/cGMP/PKG signaling in platelets, especially concerning data that this pathway is a part of the mechanism(s) which causes platelet activation. This is very important for clinical aspects and therapeutic safety because numerous drugs including nitrovasodilators, direct sGC stimulators, such as the recently developed riociguat, and PDE5 inhibitors such as dipyridamole and sildenafil, which all stimulate the cGMP/PKG pathway, are widely used clinically for treatment of different disorders. There are no indications in the literature that these drugs when clinically used activate platelets and/or cause thrombosis which would be a serious adverse effect. The recently developed quantitative phosphoproteomic methods open a new era in the field of intracellular signaling, especially related to kinase/phosphatase functions. Similar to the search for and analysis of PKA substrates in human platelets, our ongoing studies with NO donors and sGC stimulators suggest the presence of hundreds of PKG substrates in these cells, which represent a broad spectrum of intracellular signaling pathways. It is therefore clear that the effects of cGMP in platelets are not mediated by a handful of selective PKG substrates but by hundreds, which are partly specific for PKG and mostly overlap with PKA substrates. There is increasing evidence that many of these proteins function as complexes with other proteins and/or signaling networks. Future analysis of these PKG substrates and their protein complexes involved in different platelet inhibitory pathways will help to understand the mechanisms of cGMP-regulated platelet inhibition and could be a basis for the development of new antiplatelet drugs that may target only specific aspects of platelet functions.

## Data Availability

The data underlying this article will be shared at reasonable request to the corresponding author.
